# Crystal Gold Foil, Adhesive Foil, Crystal Gold

**Published:** 1858-08

**Authors:** 


Crystal Gold Foil—Adhesive Foil—Crystal Gold.—The manifestly growing interest felt in these articles, makes it incumbent on us to make a few remarks.

Until recently it has been the aim of nearly all Foil makers to *overcome* the *adhesiveness*, or in other words, to make it non-adhesive, simply because, with the old style of instruments and manner of manipulating, adhesive foil could not be worked—it clogged or balled, before it could be fixed in the cavity with the smooth blunt instrument.

We have consulted with several of the best and most popular Foil makers in the country, and learn from their experience that all foil is made of *pure gold*, that when freshly made, it is so adhesive, that if one leaf falls upon another, they can not be separated without tearing, that after exposure to the atmosphere, a short time, it absorbs moisture and becomes soft and pliable or non-adhesive, just in proportion to the length of time it has been exposed—to *restore* the adhesiveness it is only necessary to heat it over a spirit lamp sufficient to produce *perfect dryness*.

The special claim set up by A. J. Watts & Co., for what they are *pleased* to call "crystal Gold Foil," has created quite a revolution in materials and INSTRUMENTS for filling teeth, but more especially in *instruments*. Arthur's patterns, Blakesley's patterns, Taft's patterns, and others with finely serated points, gives the operator advantages never before possessed, it enables him to use the soft or non-adhesive Foil to a greater advantage than before, whilst with adhesive Foil—using time labor and skill—better fillings can be made than were before anticipated or attempted—the great *merit* is in the *instruments* and not in the materials; the materials which a few years ago was deemed unfit for use, is now found to make in certain cases the best of work. In adhesive foil there is *nothing new*, but in the *instruments* and *skill* to use it to advantage, much progress has been made.

That "Crystal Gold Foil" is made of "Crystal Gold," hence its name, that thus at all times and under all circumstances pure gold can be produced with perfect uniformity, which can be done in no other way, is *assuming* that all the chemical and metalurgical science and skill is concentrated in one establishment, the ridiculousness of such a proposition must be apparent to every Dentist who has any pretention to literary acquirements.

When Crystal Gold was first introduced by A. J. Watts & Co., it was sold at $32 per oz. for less than one ounce, and $30 per oz., when 1 oz. or more was taken. After considerable experiment and trial, the uncertainty of result, at one time, drove it almost entirely out of market, then commenced the manufacture of Crystal Gold Foil, the retail price of which was fixed at $32 per oz. A few operators still insisted on having Crystal Gold, which could not be had, because, as alleged, it could not be afforded at the price at which it had been sold. It was then advanced to $36 per oz., and kept at that for several months; during all this time Crystal Foil was pushed upon the market at $32 whilst the "Crystal Gold," of which it is made, could not be afforded for less than $36; here is a species of *consistency* that we must confess is beyond our comprehension. Within a few weeks the market is again supplied with Crystal Gold at $3,75 per ⅛ oz. or $30 per oz.; what the next phase will be we can not anticipate. We are however, satisfied that we can supply foil of other manufactures, which if used fairly and without prejudice, can not be distinguished from Crystal Foil, even by the most zealous advocate of the latter. With all these inconsistencies, we must confess that the Foil manufactured by A. J. Watts & Co. is a *good* article; yes, *a first rate article*, being neatly and closely enveloped, it retains its adhesiveness longer than foils put up in loose books. Such of the profession as prefer the Crystal Gold or Crystal Gold Foil we would be pleased to supply. We sell all kinds alike without prejudice, what we object to is, the ridiculous assumption that they alone can produce *pure* gold with uniformity, and that the Foil is made from Crystal Gold. If they had come into the market with more modesty, they would have met with greater favor.

A few years ago an article of *Crystal Gold* was made by Drs. Taft and Watt of Xenia, Ohio, which by many was esteemed far superior to any other, it was more dense, and in distinct and definite crystals, requiring less time and less labor, to produce the same effect. We have heard it rumored, that a gentleman familiar with their mode intends to supply the market at $32 per oz.; if this is done, a lively competition may be expected.

